# ^1^H NMR metabolic profiling of *Staphylococcus pseudintermedius* isolated from canine uroliths

**DOI:** 10.1371/journal.pone.0277808

**Published:** 2022-11-17

**Authors:** Nahathai Uttamamul, Manida Suksawat, Jutarop Phetcharaburanin, Supranee Jitpean, Aroonlug Lulitanond, Nattaya Sae-ung, Patcharee Boonsiri, Ratree Tavichakorntrakool

**Affiliations:** 1 Centre for Research and Development of Medical Diagnostic Laboratories, Faculty of Associated Medical Sciences, Khon Kaen University, Khon Kaen, Thailand; 2 School of Medical Technology, Faculty of Associated Medical Sciences, Khon Kaen University, Khon Kaen, Thailand; 3 Khon Kaen University International Phenome Laboratory, Khon Kaen University, Khon Kaen, Thailand; 4 Cholangiocarcinoma Research Institute, Faculty of Medicine, Khon Kaen University, Khon Kaen, Thailand; 5 Division of Surgery, Faculty of Veterinary Medicine, Khon Kaen University, Khon Kaen, Thailand; 6 Department of Biochemistry, Faculty of Medicine, Khon Kaen University, Khon Kaen, Thailand; Fisheries and Oceans Canada, CANADA

## Abstract

*Staphylococcus pseudintermedius* is a urease-producing bacteria which is a major cause of magnesium ammonium phosphate (MAP) urolithiasis in canine. A positive urolith culture is an important risk factor for MAP urolithiasis in canine. The mechanism underlying the metabolic changes of *S*. *pseudintermedius* after crystallization in artificial urine (AU) needs more defined baseline metabolic information. Therefore, we extensively investigated the metabolic changes of *S*. *pseudintermedius* extensively after crystallization in AU. A high urease activity and positive biofilm formation strain, entitled the *S*. *pseudintermedius* (SPMAP09) strain, was isolated from canine MAP uroliths, and analyzed using nuclear magnetic resonance (NMR) spectroscopy-based metabolomics. The molecular mechanism-specific metabolic phenotypes were clearly observed after crystallization in AU at day 3. The crystals induced by SPMAP09 were also confirmed and the major chemical composition identified as struvite. Interestingly, our findings demonstrated that a total of 11 identified metabolites were significantly changed. The levels of formate, homocarnosine, tyrosine, cis-aconitate, glycolate, ethyl malonate, valine and acetate level were significantly higher, accompanied with decreased levels of inosine, glucose, and threonine at day 3 compared with the initial time-point (day 0). In addition, our results exhibited that the glyoxylate and dicarboxylate metabolism was significantly related to the SPMAP09 strain at day 3 in AU. Thus, metabolic changes of the SPMAP09 strain after crystallization in AU potentially helps to explain the preliminary molecular mechanism for the crystals induced by *S*. *pseudintermedius*.

## Introduction

Magnesium ammonium phosphate (MAP) urolithiasis is attributed to urease-producing bacteria [1, 2]. *Staphylococcus* spp. (urease-producing bacteria) is the most common causative bacteria found in urine and uroliths of the canine [3, 4]. Intriguingly, our previous data [5] indicated that *S*. *pseudintermedius* was the most common causative bacteria found in the nidus of canine MAP uroliths. A positive urolith culture is an important risk factor for MAP urolithiasis in the canine. Based on their locales in the urolith nidus [6], we hypothesized that the microorganisms found in the urolith nidus was indeed the causative bacteria involved in the stone formation and pathogenesis. Bacterial urease splits urea and promotes the formation of ammonia and carbon dioxide, resulting in increased levels of ammonia in urine. Ammonia combines with hydrogen ions to produce ammonium ions. Buffering of hydrogen ion by ammonia results in urine alkalinization and a shift of phosphorus from H_3_PO_4_ to PO_4_^3–^, leading to increased levels of NH_4_^+^ and PO_4_^3–^ in the presence of Mg^2+^ and an alkaline pH, the solubility and formation products of struvite may be exceeded, resulting in precipitation [7]. Several approaches have been developed to explore the molecular mechanisms of *S*. *pseudintermedius* induced crystals which need more information. Therefore, we investigated the metabolic changes of SPMAP09 strain after crystallization in AU extensively. A high urease activity and positive biofilm formation strain entitled *S*. *pseudintermedius* isolated from canine MAP uroliths was selected and analyzed using NMR-based metabolomics.

## Materials and methods

### Source of bacterial strain

This study was approved by the Animal Ethics Committee of Khon Kaen University, Khon Kaen, Thailand (ACUC-KKU-25/2560). Urolith samples were collected from a total of 56 canines with urolithiasis and were later cultured for bacteria. Inclusion and exclusion criteria of sample collection, and antimicrobial susceptibility tests were described in our previous report [5]. Among them, 27 MAP uroliths were positive for bacterial culture. Intriguingly, *S*. *pseudintermedius* was the most common bacteria found in MAP uroliths. Note that the selection of the SPMAP09 strain, including the demographic characteristics and clinical chemistry data of the canine (sex, age, breed, urine pH, urinalysis, urolith size, and chemical composition of the urolith), multidrug resistance, high level of biofilm and urease activity were used. Therefore, only the one SPMAP09 strain was prepared for further analysis.

### Crystallization and chemical compositions of crystals induced by SPMAP09

Artificial urine (AU) for crystallization was prepared by modified AU-Siriraj [8] with 7.889 g/L of Luria-Bertani (LB) broth (Difco, France). SPMAP09 was cultured on blood agar at 35–37°C for 18–24 h. A colony of bacteria was grown in LB broth at 35–37°C for 18–24 h. The concentration of bacteria to the 0.5 McFarland standard was adjusted to 1:100 and then 150 μL of the bacterial suspension added to 1,350 μL of AU to a final concentration of 10^5^ CFU/mL followed by incubation at 35–37°C for three days. The initial early stage of crystallization was observed using an inverted microscope within three days. AU and *Proteus mirabilis* isolated from the stone matrix were used as negative and positive controls [6, 9], respectively. The pH values of crystal formation were also determined at day 0, 1, 2 and 3 using a pH meter (Humming Probe pH Measurement system, UltraE Co. Ltd., Taiwan). Crystallization and pH experiments were repeated five times. The chemical composition of the crystal induced by bacteria was confirmed by Tensor-II Attenuated Total Reflection Fourier-transform infrared spectrometer (ATR-FTIR) (Bruker Optic, Germany). After centrifugation (1,000g, 4°C for 15 min), the samples without preservative agent and washing step were analyzed by ATR-FTIR. Ten microliters (μL) of AU sediment were applied onto a diamond crystal attenuated total reflectance accessory and air dried for 25 min. After that, infrared spectra were recorded at the wave numbers from 4000 to 400 cm^-1^ at 4 cm^-1^ resolutions, accumulating 64 scans per spectrum. Each sample was measured three times and analyzed by OPUS program version 8.2.28 for Windows (Bruker Optic GmBH, Germany). In addition, FTIR results were calculated and compared to the Bruker kidney stone library.

### Sample preparation and ^1^H NMR spectroscopic analysis

To compare metabolic profiles of AU containing *S*. *pseudintermedius* after crystallization in AU at day 3 and the initial time-point (day 0), SPMAP09 was suspended in AU to a concentration of 10^5^ CFU/mL at 37°C for day 0 and day 3. Each AU sample included five replicates. The AU samples were centrifuged at 13,000 rpm for 10 min. A total of 540 μL of supernatant was mixed with 60 μL urine buffer containing 1.5M KH_2_PO_4_, 2mM NaN_3_ and 1% TSP as internal standard. Then, the mixture was centrifuged at 12,000 rpm at 4°C for 19 min and 580 μL of supernatant was transferred into a NMR tube with 5 mm diameter for metabolic profiling. ^1^H NMR spectra were acquired using a 400 MHz NMR spectrometer (Bruker, USA) with CryoProbe Prodigy and the Carr−Purcell−Meiboom−Gill (CPMG) pulse sequence [RD−90˚−(τ−180˚−τ)n−acquisition] was applied to analyze the AU samples at 310 K in 64 scans [10].

### NMR spectral data pre-processing and metabolite identification

The baseline correction and phasing of NMR spectra were performed with TopSpin software (Bruker, USA) for chemical shift referencing. NMR spectral data were processed using MATLAB R2015 version 9.10 (MathWorks Inc., USA) software equipped with the IMPaCTS toolbox (https://doi.org/10.5281/zenodo.3077413) for conducting probabilistic quotient normalization (PQN). Statistical total correlation spectroscopy (STOCY) [11] was used to validate the appearances of correlated resonances on 1-dimensional NMR spectra which were searched for against public databases including the human metabolome database (HMDB) and ChenomxNMR Suite version 9.0 (Chenomx Inc., Canada) for metabolite identification. Data can be accessed at Open Science Framework (https://osf.io/sqftm/).

### Statistical analysis

Multivariate statistical analysis was performed with the MATLAB 2015 version 9.10 environment equipped with IMPaCTS toolbox (https://doi.org/10.5281/zenodo.3077413). Pre-processed spectral data were imported into SIMCA for conducting principal component analysis (PCA) and orthogonal signal correction-projection to latent structures discriminant analysis (O-PLS-DA) with a pareto scaling method. All O-PLS-DA models were constructed based on one predictive component and one orthogonal component. The O-PLS-DA scores and coefficient plots were also obtained. The red-blue color spectrum represents the correlation coefficient values |r|. The red color indicates higher correlation while the blue color indicates lower correlation of the variables. Benjamini-Hochberg false discovery rate correction was also applied for differential biomarker discovery. The goodness of fit and predictability of the models were determined by R^2^ and Q^2^ values, respectively. The validation of all models involved in this experiment was assessed using cross-validation and permutation *p*-value (*p* < 0.05).

### Pathway analysis

Maximum intensities of selected metabolites were further used for pathway impact analysis using MetaboAnalyst [12] based on the Kyoto Encyclopedia of Genes and Genomes (KEGG) pathway library of prokaryotes to investigate the impact of different metabolic profiles of the SPMAP09 strain after crystallization in AU at day 3 compared with the initial point (day 0). The cut-off values of 0.1 for pathway impact score, *p* < 0.05 and false discovery rates < 0.25 were applied to cover the confidence of practice and to filter insignificant pathways [13].

## Results

### Crystallization by SPMAP09

**Fig 1:**
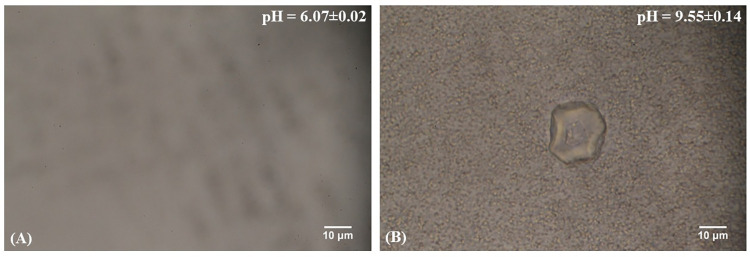
Image of crystals induced by SPMAP09 in AU at day 0 (A) and day 3 (B) (400X and scale bar,10 μM).

The process of crystallization in AU was induced by SPMAP09 to mimic the real urinary tract infection (UTI), which may relate to urinary stone genesis. The results demonstrate that the crystals were observed in AU with SPMAP09 strain at day 3 (**Fig 1**). The results show that the urinary pH (mean±SD) was increased from 6.07±0.02 to 9.55±0.14. The crystals could be observed in the positive control (*P*. *mirabilis*) but not in the negative control (AU). For the crystallization in the presence of SPMAP909, an irregular shape of crystal was observed, and the pH of AU was changed to 9.55 at day 3.

### Chemical compositions of crystals induced by SPMAP09

**Fig 2:**
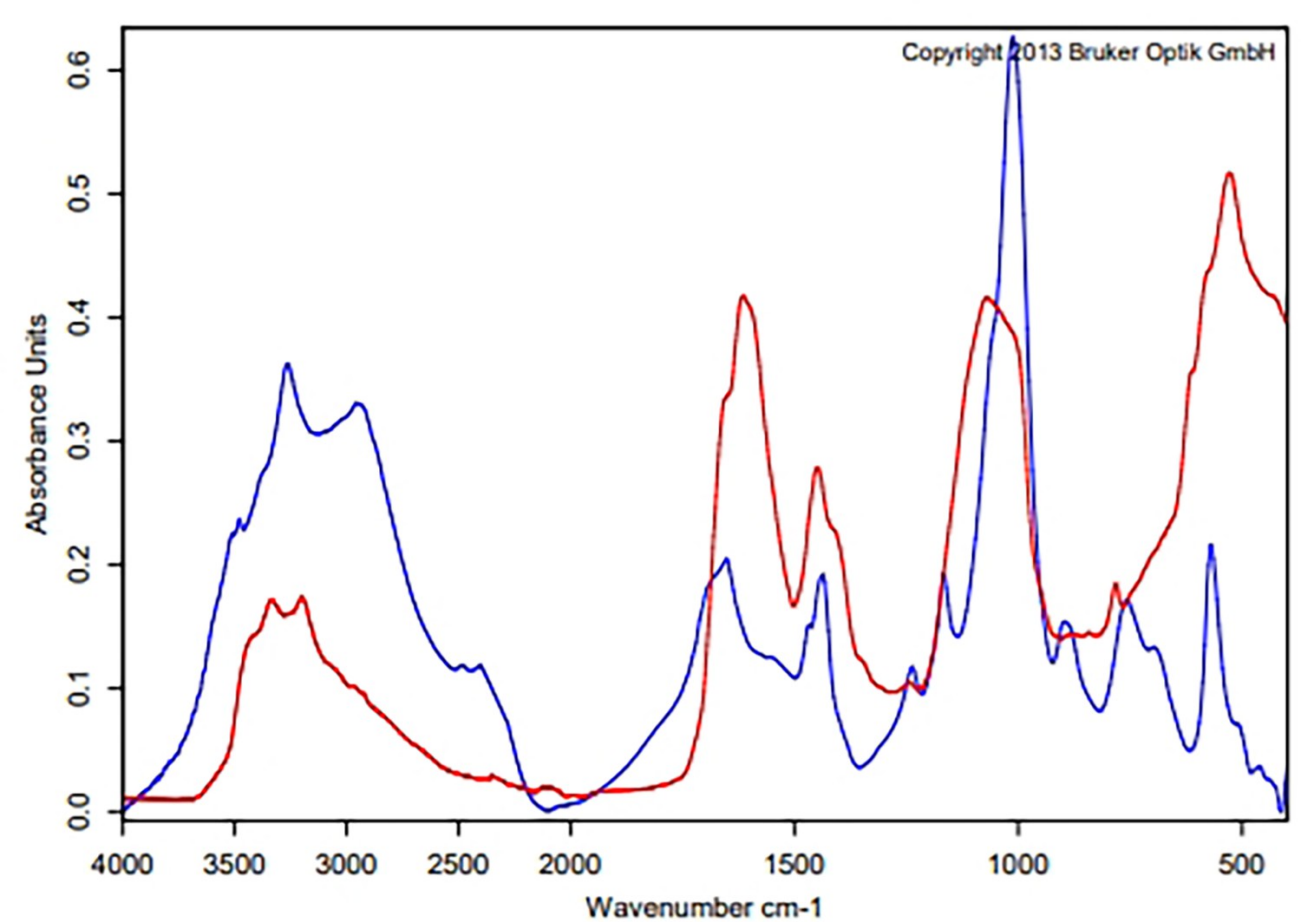
Search for FTIR spectrum result of crystals induced by SPMAP09 (red curve) with Bruker kidney stone library (blue curve). The main component of the crystal was identified as struvite.

The crystals induced by SPMAP09 at day 3 were confirmed and the chemical compositions were analyzed by the ATR-FTIR spectroscopic method (**Fig 2**). The analysis of induced crystal samples (red curve) with struvite (magnesium ammonium phosphate) as the main component (60%) was a hit quality point (blue curve). Additionally, the substances newberyite (dimagnesium phosphate trihydrate) and protein were both detected (20%).

### Metabolic profiling of SPMAP09 after crystallization in artificial urine

**Fig 3:**
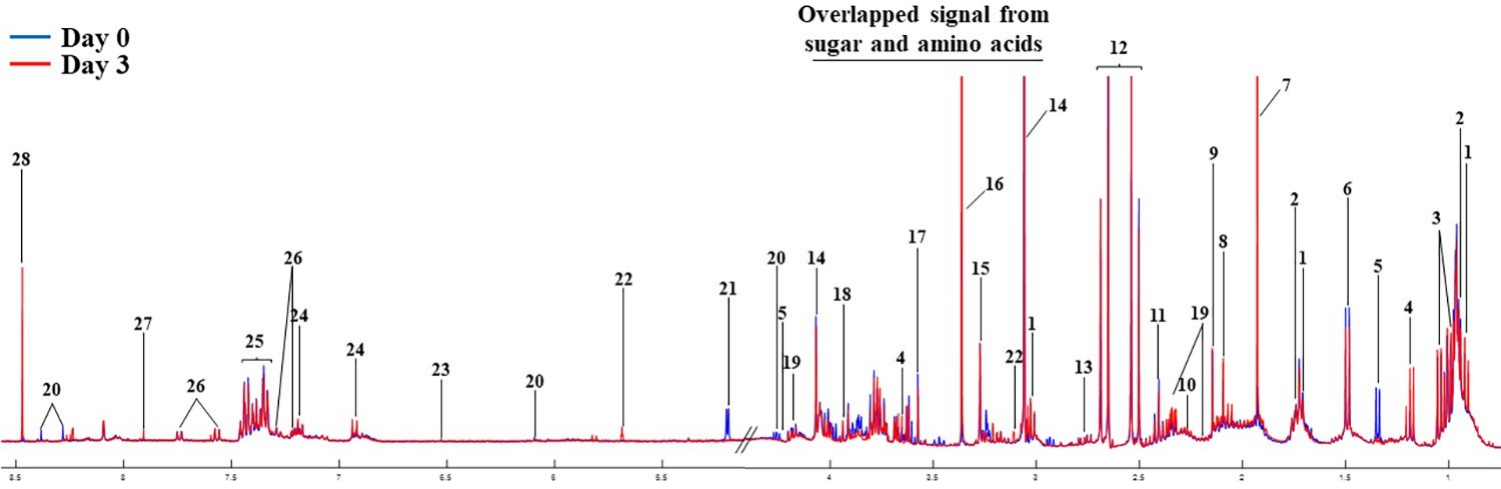
Representative median ^1^H NMR CPMG spectra of SPMAP09 strain after crystallization in AU observed at initial point and 72 h. The full metabolite list is presented in Table 1.

The metabolic profiles of the SPMAP09 strain after crystallization in AU at day 3 compared to the initial point (day 0) were analyzed using NMR-based metabolomics. Spectral data were then acquired and pre-processed. A total of 28 metabolites with the median ^1^H NMR CPMG spectra were identified in SPMAP09 after crystallization in AU (**Fig 3** and **Table 1)**.

**Table 1:** List of identified metabolites in the SPMAP09 strain after crystallization in AU.

No.	Metabolite	^1^H NMR chemical shift
1	Ethylmalomate	0.9055 (t), 1.708 (m), 3.01 (t)
2	Leucine	0.9426 (t), 1.723 (m), 3.725 (t)
3	Valine	0.99 (d), 1.037 (d), 2.279 (dh), 3.629 (d)
4	Ethanol	1.1707 (t), 3.634 (q)
5	Threonine	1.3362 (d), 3.605 (d), 4.2045 (m)
6	Alanine	1.4825 (d), 3.777 (q)
7	Acetate	1.9286 (s)
8	Unknown 1	2.0938 (s)
9	Unknown 2	2.147 (s)
10	Unknown 3	2.3226 (m)
11	Succinate	2.406 (s)
12	Citrate	2.503 (d), 2.651 (d)
13	Unknown 4	2.7347 (dd), 2.828 (d)
14	Creatinine	3.0591 (s), 4.069 (s)
15	Trimethylamine *N*-oxide	3.2737 (s)
16	Methanol	3.3622 (s)
17	Glycine	3.575 (s)
18	Glycolate	3.9388 (s)
19	Proline	2.032 (m), 2.36 (m), 3.3806 (m), 4.1673 (dd)
20	Inosine	3.807 (dd), 3.887 (dd), 4.245 (q), 6.086 (d), 8.28 (s), 8.384 (s)
21	Glucose	3.277 (dd), 3.395 (m), 3.448 (m), 3.544 (dd), 3.725 (m), 3.809 (m), 3.904 (dd), 5.193 (d)
22	Cis-aconitate	5.6876 (s), 3.108 (d)
23	Fumarate	6.527 (s)
24	Tyrosine	3.402 (dd), 3.211 (dd), 3.939 (dd), 6.9176 (d), 7.191 (d)
25	Phenylalanine	3.136 (dd), 3.284 (dd), 3.995 (dd), 7.3338 (m)
26	Tryptophan	3.293 (dd), 3.494 (dd), 4.042 (dd), 7.203 (t), 7.291 (t), 7.556 (d), 7.7321 (d)
27	Homocarnosine	1.88 (m), 2.375 (m), 2.917 (m), 2.959 (dd), 3.173 (dd), 3.211 (t), 7.9077 (s)
28	Formate	8.4709 (s)

Key: s, singlet; bs, broad singlet; d, doublet; t, triplet; m, multiplet; q, quadruplet; dd, double doublet.

### Metabolic adaptation of SPMAP09 after crystallization in artificial urine

**Fig 4:**
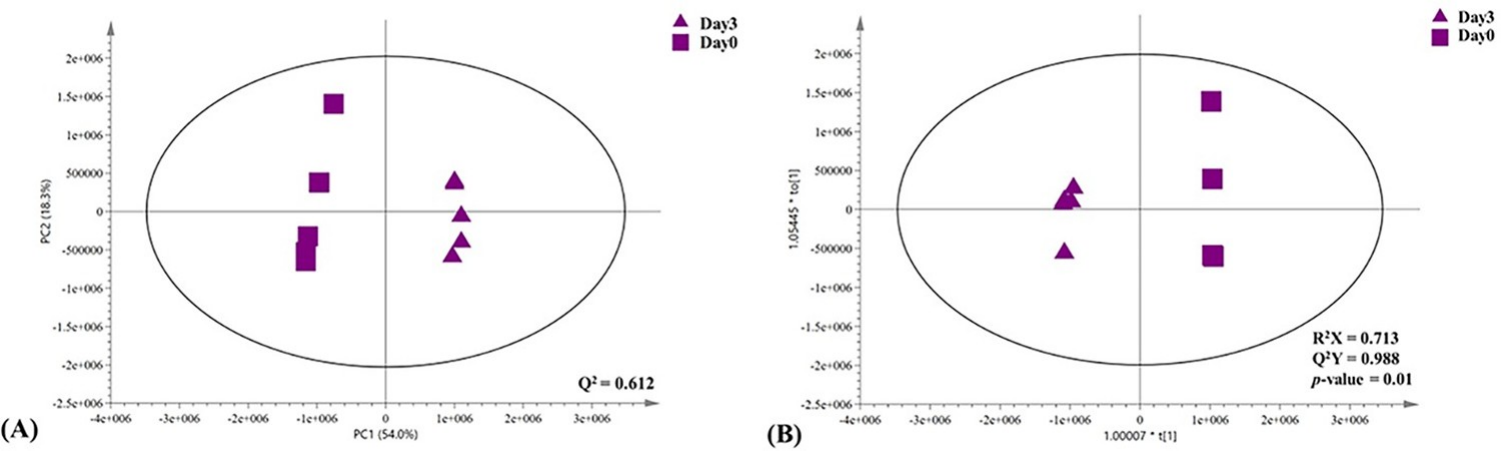
Overview of metabolic similarities and differences metabolic profiles from SPMAP09 strain after crystallization in AU observed at day 0 (squares) and day 3 (triangles); (A) PCA score plots and (B) O-PLS-DA score plots.

**Fig 5:**
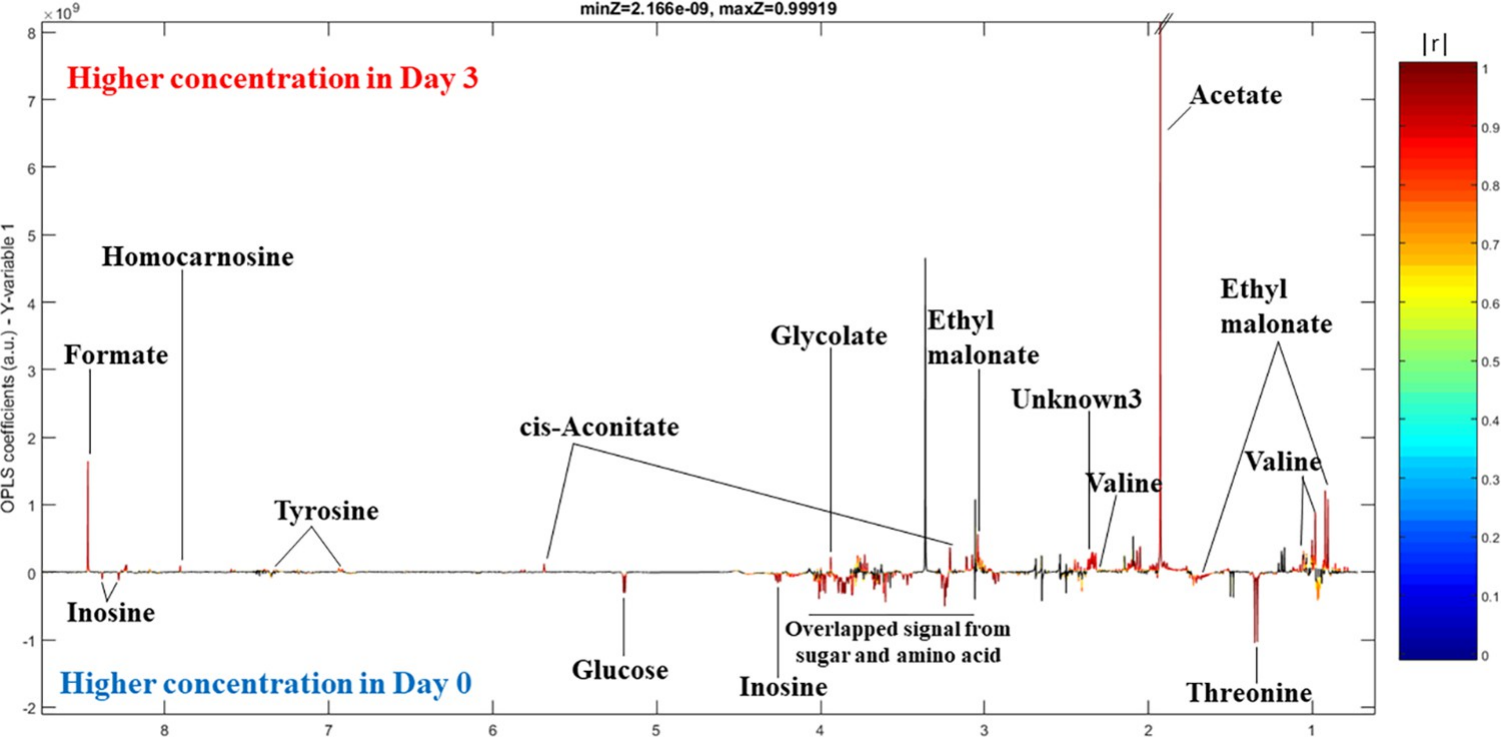
O-PLS-DA loading plots displaying significantly changes in SPMAP09 strain after crystallization in AU observed at day 0 and day 3.

The metabolic overview, similarities, and differences of samples were evaluated by principal component analysis (PCA). The PCA scores plot revealed a clear class separation between day 0 and day 3 (**Fig 4A**: PC1 = 54.0% and PC2 = 18.3%; Q^2^ = 0.612). Orthogonal partial-least square discriminant analysis (O-PLS-DA) was performed based on Pareto-scaled data to explore the metabolic changes induced by the SPMAP09 strain. In **Fig 4B**, a goodness of fit (R^2^X) and a goodness of prediction (Q^2^Y) for the SPMAP09 model were used to assess the quality of the statistical models for which a cut-off value above 0.5 was considered acceptable for the classification model for biological samples [14]. Furthermore, the CV-ANOVA *p*-value <0.05 was used to demonstrate the model validity. O-PLS-DA (R^2^X = 0.713, Q^2^Y = 0.988, *p*-value = 0.01) score plots of the pairwise comparison model between day 0 and day 3 groups demonstrated distinct class separation (**Fig 4B**). Moreover, the O-PLS-DA corresponding loading plot showed increased relative concentrations of formate, homocarnosine, tyrosine, cis-aconitate, glycolate, ethyl malonate, valine and acetate at day 3, while relative concentrations of inosine, glucose and threonine were significantly decreased in day 3 group (**Fig 5**).

**Fig 6:**
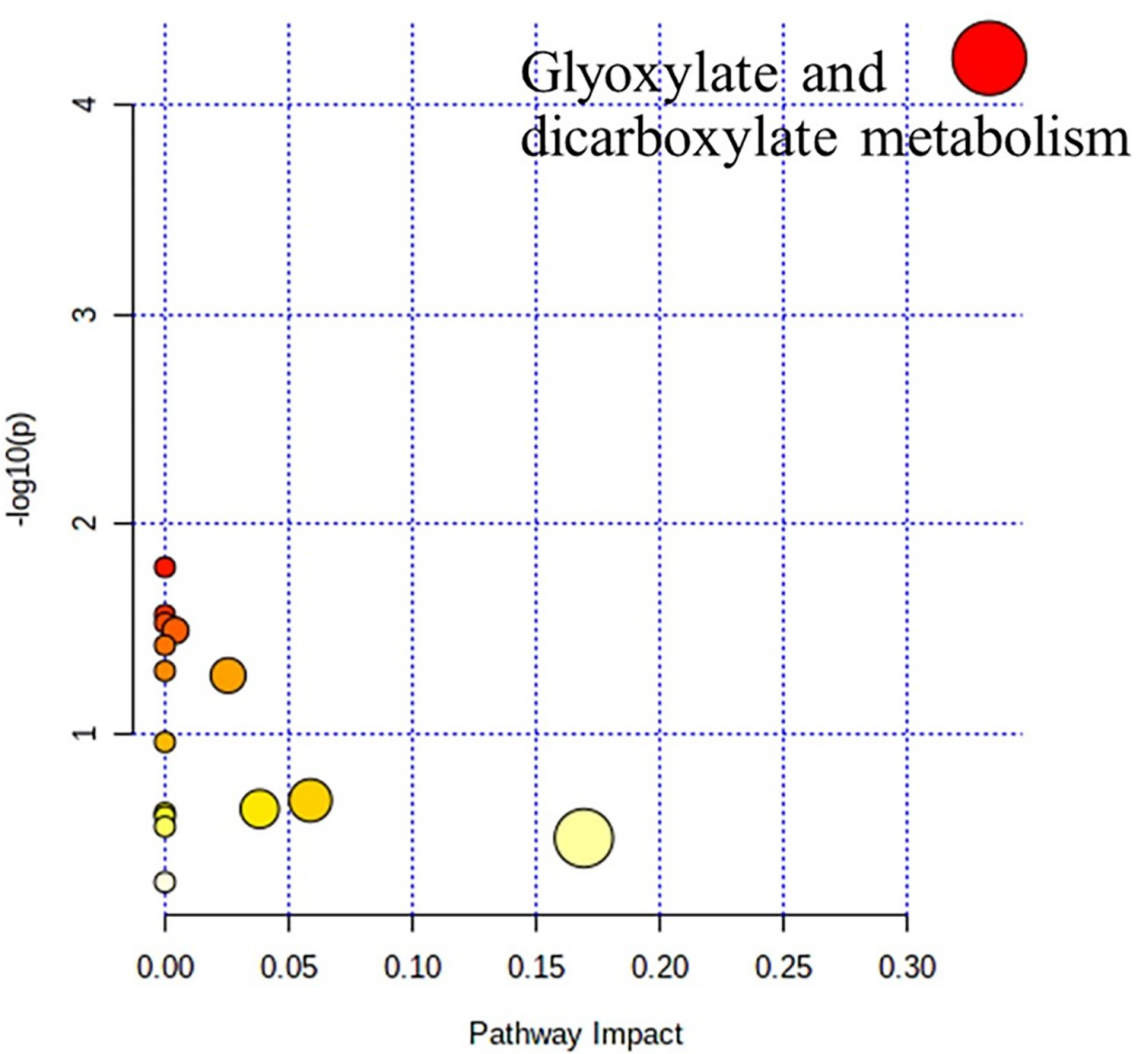
Graphical overview for the metabolome viewing of the pathway analysis. The X-axis indicates the impacts on pathway, while the Y-axis indicates the significance of changes (-log (*p*)) in the pathway by the identified metabolites represented by different color intensities.

Pathway analysis of significantly contributing metabolites was performed using MetaboAnalyst based on the KEGG pathway of prokaryotes database. Cut-off values for pathway impact score > 0.1, false discovery rate < 0.25 and *p* < 0.05. The figure was obtained from MetaboAnalyst using our experimental data as input. Our findings also demonstrate that only glyoxylate and dicarboxylate metabolisms corresponded to SPMAP09 after crystallization in AU at day 3 (**Fig 6** and **Table 2**). Four metabolites including formate, cis-aconitate, glycolate and acetate were involved with the glyoxylate and dicarboxylate metabolism.

**Table 2:** Metabolic pathway analysis of the SPMAP09 strain after crystallization in AU observed at initial point (day 0) and day 3.

Metabolic pathway	Total Cmpds.	Hits	Raw *p*	-LOG(*p*)	Holm adjusted	FDR	Impact
Glyoxylate and dicarboxylate metabolism	20	4	0.0000593	4.2272	0.0044449	**0.0044449**	**0.33333**
Aminoacyl-tRNA biosynthesis	45	3	0.0160360	1.7949	1	0.4734300	0
Methane metabolism	21	2	0.0270960	1.5671	1	0.4734300	0
Valine, leucine and isoleucine biosynthesis	22	2	0.0296020	1.5287	1	0.4734300	0
Pyruvate metabolism	23	2	0.0322000	1.4921	1	0.4734300	0.00426
Novobiocin biosynthesis	3	1	0.0378750	1.4217	1	0.4734300	0
Tyrosine metabolism	4	1	0.0502100	1.2992	1	0.4946900	0
Glycolysis / gluconeogenesis	30	2	0.0527670	1.2776	1	0.4946900	0.02555
Taurine and hypotaurine metabolism	9	1	0.1097800	0.9595	1	0.9148000	0
Sulfur metabolism	18	1	0.2085800	0.6807	1	1.0	0.05882
Citrate cycle (TCA cycle)	20	1	0.2291500	0.6399	1	1.0	0.03831
Pantothenate and CoA biosynthesis	21	1	0.2392600	0.6211	1	1.0	0
Phenylalanine, tyrosine and tryptophan biosynthesis	22	1	0.2492400	0.6034	1	1.0	0
Valine, leucine and isoleucine degradation	25	1	0.2784900	0.5552	1	1.0	0
Glycine, serine and threonine metabolism	29	1	0.3158900	0.5005	1	1.0	0.16939
Purine metabolism	54	1	0.5127000	0.2901	1	1.0	0

Total Cmpds., the total number of compounds in the pathway; Hits, the matched metabolites uploaded to MetaboAnalyst; Raw *p*, the original *p*-value calculated from the enrichment analysis; Holm adjusted, *p*-value adjusted by the Holm-Bonferroni method; FDR, the *p*-value adjusted by the false discovery rate; Impact, the pathway impact value calculated from pathway topology analysis.

## Discussion

Our previous data [5] showed that *S*. *pseudintermedius* was the most common causative bacteria found in the nidus of canine MAP uroliths. Based on *S*. *pseudintermedius* in the stone nidus, we hypothesized that it was involved in stone formation and pathogenesis. This study shows that the SPMAP09 strain could induce struvite crystals (**Figs 1 and 2**) at the urine pH 9.55. The solubility and formation products of struvite may be exceeded in alkaline urine, resulting in precipitation [7, 15]. A high urease activity and positive biofilm formation strain entitled SPMAP09 strain isolated from canine MAP uroliths was selected and analyzed by NMR. We investigated the changes of metabolic profiles from the SPMAP09 strain after crystallization in AU at day 3, compared with the initial point. Our findings demonstrate that the increased relative concentrations of formate, homocarnosine, tyrosine, cis-aconitate, glycolate, ethyl malonate, valine and acetate at day 3, while relative concentrations of inosine, glucose and threonine decreased significantly. Only glyoxylate and dicarboxylate metabolism corresponded to SPMAP09 after crystallization in AU at day 3. Our results were consistent with a recent study showing that glyoxylate and dicarboxylate metabolism was found in the metabolic profiling from the urine of kidney stone formers by ^1^H NMR spectroscopy-based metabolomics [16]. The matched metabolites (formate, cis-aconitate, glycolate and acetate) were related with the glyoxylate and dicarboxylate metabolism. The increase of formate may be influenced by biofilm and urease activity. A study by Resch et al. [17] has revealed that urease genes and genes encoding proteins of *Staphylococcus aureus* involved in the synthesis of formate were upregulated in biofilms, compared to planktonic cells. Moreover, the urease activity may be a factor to balance the cells under low pH conditions [18, 19]. In addition, cis-aconitate may response to aconitase as a bifunctional protein which may have a regulatory role in *S*. *aureus* virulence factor production [20], while the decreasing levels of inosine, glucose and threonine may result from the growth process. Based on these results, when the concentration of glucose decreased, an accumulation of growth-inhibitory molecules such as acetate may occur [20, 21]. It is noticeable that glyoxylate and dicarboxylate metabolism corresponded significantly to the SPMAP09 strain after crystallization in AU. This study gave a preliminary explain on the molecular mechanism of crystals induced by *S*. *pseudintermedius*, through the use of ^1^H NMR spectroscopy-based metabolomics. However, the mechanism of this metabolic pathway and altered metabolites involved in *S*. *pseudintermedius*-induced crystals remains unclear, but our information provides some new points for further investigation.

## Conclusion

Our new findings have revealed an exploratory metabolic profiling of AU containing *S*. *pseudintermedius* after struvite crystallization via ^1^H NMR spectroscopy-based metabolomics. The glyoxylate and dicarboxylate metabolism was considered having the highest impact in response to the struvite crystallization. Our findings may lead to rethinking future research regarding the causative role of *S*. *pseudintermedius*-induced crystals in canine urolithiasis. Nevertheless, the roles of *S*. *pseudintermedius* in stone formation should be verified and elucidated by an *in vivo* study.
